# Genome Sequences of Five Microbacterium foliorum Phages, GaeCeo, NeumannU, Eightball, Chivey, and Hiddenleaf

**DOI:** 10.1128/mra.01106-22

**Published:** 2023-03-02

**Authors:** Matthew D. Mastropaolo, Patricia C. Fallest-Strobl, DaeKwon M. Sequira, Davia D. Campbell, Christopher J. Negro, Kho S. Tuang, Ian M. Sigmund-Hamre, Courtney L. Womack, Thomas Mansbridge, Amanda L. Metzler, Emily G. Sasher, Colleen Collins, Nicholas K. Crowley, Victoria R. Dower, Megan Bates, Christian Bjorkelo, Hailey Johnson, Lauren R. Salvitti, Neumann University Phage Discovery Group

**Affiliations:** a Mathematics and Sciences, Neumann University, Aston, Pennsylvania, USA; b Charles River Laboratories, Newark, Delaware, USA; Queens College Department of Biology

## Abstract

Five siphoviruses were isolated from soil in southeastern Pennsylvania using Microbacterium foliorum. Bacteriophages NeumannU and Eightball have 25 predicted genes, Chivey and Hiddenleaf have 87 genes, and GaeCeo has 60 genes. Based on gene content similarity to sequenced actinobacteriophages, these five phages are distributed across clusters EA, EE, and EF.

## ANNOUNCEMENT

Bacteriophages are an abundant and genetically diverse group of viruses that prey on bacteria (1). Here we report on the isolation and characterization of five bacteriophages isolated using Microbacterium foliorum NRRL B-24224.

All bacteriophages were isolated from soil collected in southeastern Pennsylvania (Table 1), using standard methods (2). Soil samples were suspended in peptone-yeast extract-calcium (PYCa) liquid medium and incubated with shaking at 250 rpm for 2 h at 30°C. The wash was then collected by centrifugation and filtered through a 0.22-μm filter, and the filtrate was plated in PYCa soft agar containing M. foliorum and incubated at 30°C for up to 48 h. Each bacteriophage was purified through three rounds of plating, and plaque morphologies are presented in Table 1. Bacteriophage morphology was determined by negative-staining transmission electron microscopy (TEM), and measurements of the capsids and tails were determined manually for a minimum of three particles. All isolated bacteriophages are siphoviruses (Table 1).

**TABLE 1:** Bacteriophage, plaque morphology, and genomic characteristics^*a*^

Phage name	Soil sample collection site	Isolation yr	Plaque morphology	Plaque size^*b*^ (mm)	Capsid size^*c*^ (nm)	Tail length^*c*^ (nm)	Approx shotgun coverage (fold)	Genome length (bp)	Genome end characteristic	G+C content (%)	No. of ORFs^*d*^	No. of tRNAs	Cluster
Chivey	Garnet Valley, PA, 39.853972 N, 75.479417 W	2018	Clear middle, cloudy halo^*e*^	1–1.5	59–77	154–181	2,383	56,082	Circularly permutated	63.7	84	0	EF
Hiddenleaf	Garnet Valley, PA, 39.853972 N, 75.479417 W	2018	Small and clear	2	68	150–168	1,334	56,082	Circularly permutated	63.7	84	0	EF
Eightball	Chester, PA, 39.841972 N, 75.389932 W	2020	Small and clear	2	ND	ND	5,973	17,439	3′ single-stranded overhang, 5′-CCCGCCCCA-3′	68.7	25	0	EE
NeumannU	Aston, PA, 39.874167 N, 75.440889 W	2018	Small and clear	3–4	41	100	6,452	17,445	3′ single-stranded overhang, 5′-CCCGCCCCA-3′	68.7	25	0	EE
GaeCeo	Aston, PA, 39.871102 N, 75.436438 W	2018	Small and cloudy	0.9	50–58	134–146	2,726	40,168	Circularly permutated	63.4	60	1	EA^*f*^

a “ND” indicates the TEM was not performed.

b Based on the size of a minimum of 3 measured plaques.

c Based on the measurements of a minimum of 3 particles from a TEM.

d ORFs, open reading frames.

e Indicates a clear middle of the plaque with a diffuse or cloudy edge.

f Subcluster EA9.

Genomic DNA was isolated from phage lysates using a ZnCl_2_ precipitation method as previously described (2, 3). The DNA was prepared for sequencing using the NEBNext Ultra II FS kit (New England BioLabs) and sequenced using Illumina MiSeq (v3 reagents), yielding ~200,000 single-end 150-base reads. Untrimmed reads were assembled and then checked for completeness using Newbler v2.9 (4) and Consed v29 (5), respectively (6). Phages were assigned to clusters (Table 1) based on at least 35% gene content similarity to sequences in the actinobacteriophage database, phagesDB (7, 8).

**FIG 1:**
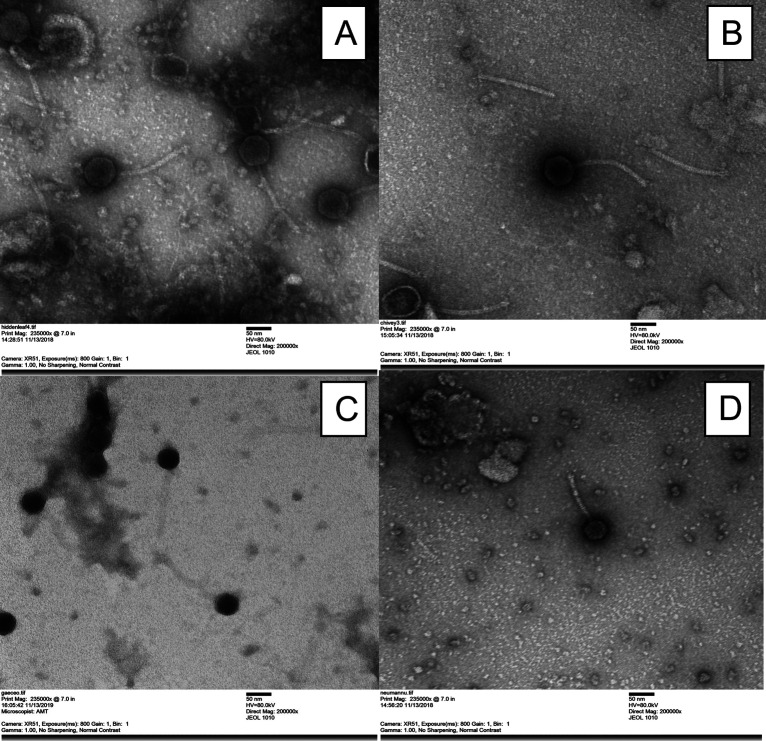
Transmission electron micrographs of bacteriophage morphology. (A) Hiddenleaf, cluster EF; (B) Chivey, cluster EF; (C) GaeCeo, cluster EA9; (D) NeumannU, cluster EE.

Initial autoannotations of the genome were performed using DNA Master v5.23.6 (http://cobamide2.bio.pitt.edu/computer.htm) embedded with GeneMark v4.28 (9) and Glimmer v3.02b (10) and then refined using Phage Evidence Collection and Annotation Network v20211202 (PECAAN [https://pecaan.kbrinsgd.org/index.html]), Phamerator (11), and Starterator v462 (https://github.com/SEA-PHAGES/starterator). Transmembrane helices were predicted using TMHMM v2.0 (12), DeepTMHMM v1.0.11 (13), TOPCONS v2.0 (https://topcons.cbr.su.se/pred/) (14), and SOSUI v1.11 (15). tRNAs were predicted using ARAGORN v1.2.41 (16) and tRNAscanSE v2.0 (17), and functional assignments were made using BLASTP v2.9 (18) and HHPRED v3.2 (19). All annotations were performed with default parameters. Genome characteristics of each bacteriophage are listed in Table 1, and the bacteriophage morphology is shown in Fig. 1.

NeumannU and Eightball are highly similar, sharing 99.9964% nucleotide identity, and contain 25 predicted genes, of which 22 are transcribed rightwards. The 3 genes that are transcribed leftward (genes 20 to 22) encode DNA-binding proteins. Hiddenleaf and Chivey also share 99.5% nucleotide identity, with all 84 predicted genes transcribed rightwards. GaeCeo has 60 predicted genes, including 1 tRNA tRNAPro, with structure, assembly, and lysis genes occupying the left half of the genome (genes 2 to 27) and transcribed rightwards and DNA metabolism genes (genes 34 to 51) occupying the right half of the genome and transcribed leftwards, with the exception of the rightmost gene (gene 60), which is transcribed rightwards. None of the five phages encode identifiable immunity repressor or integrase functions, and they are therefore likely to be lytic, consistent with the life cycle of other phages in these clusters.

### Data availability.

All genomes, NeumannU, Eightball, Chivey, Hiddenleaf, and GaeCeo, are available at GenBank under accession no. MT657332, OK040783, MT684591, MN497954, and MT657343 and Sequence Read Archive (SRA) no. SRX15940725, SRX15940721, SRX15940720, SRX15940723, and SRX15940722, respectively.
